# Controllable Strategy of Metal–Organic Framework Structural Stability: Regulation of Ligand Electronegativity by Esterification

**DOI:** 10.1002/advs.202413853

**Published:** 2024-12-05

**Authors:** Guanjie Huang, Jianzhong Ma, Jie Chen, Wenbo Zhang, Qianqian Fan, Buxing Han

**Affiliations:** ^1^ College of Bioresources Chemical and Materials Engineering Shaanxi University of Science & Technology (SUST) Xi'an 710021 China; ^2^ Beijing National Laboratory for Molecular Sciences Chinese Academy of Sciences (CAS) Beijing 100190 China

**Keywords:** collagen modification, green chemicals, metal‐organic framework

## Abstract

Structural stability of metal–organic framework (MOF) is crucial for their application, and thus it is of great significance to construct MOFs with controllable structural stability. Herein, a strategy based on adjusting the electronic environment of ligands to regulate the structure stability of MOF is proposed. Briefly, a novel Zr‐MOF (Zr‐TA) with hydroxyl groups is synthesized. The hydroxyl groups are esterified to obtain ester groups with stronger electronegativity, which can weaken the strength of coordination between metal ion and ligand, thereby regulating the structure stability of the Zr‐MOF. Notably, this strategy can achieve controllable adjustment of the structure by adding modifiers at the appropriate time. In this work, this strategy is used to greatly improving the binding ability of MOF and collagen fibers, the hydrothermal stability of crosslinked collagen fibers is enhanced by 82.6%. Surprisingly, this strategy can also be applied to other application fields that require dynamic changes in structural stability of MOF. It will open up a new pathway for controlling the structural stability and application performance of MOF.

## Graphical abstract

A new type of Zr‐MOF has been synthesized by using DL‐tartaric acid as a ligand, and the electronegativity of the ligand has been adjusted through a simple esterification of anhydride‐alcohols to precisely control the stability of the MOF structure, thereby achieving controllable permeation and binding of MOFs in collagen modification.

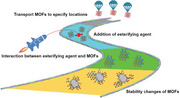

## 1 Introduction

Metal‐organic framework (MOF) is a species of porous nanomaterials with periodic network structures, generated by the coordination of metal centers and organic ligands.^[^ ^1^, ^2^, ^3^ ^]^ MOF has a set of intriguing properties, such as adjustable structure, high porosity, and large specific surface area, which have attracted widespread attention in pharmaceutical engineering, molecular separation, energy storage, synthetic catalysis, and others.^[^ ^4^, ^5^, ^6^, ^7^, ^8^, ^9^, ^10^, ^11^, ^12^, ^13^, ^14^ ^]^ In traditional cognition, MOFs with higher stability are more widely used.^[^ ^15^, ^16^, ^17^, ^18^, ^19^ ^]^ The reason is that the collapse of MOFs structure can lead to the degradation of their various functions, especially the loss of pore structure.^[^ ^20^ ^]^ Therefore, MOFs with structurally unstable characteristics are gradually being overlooked.

In fact, generous studies have shown that the instability of MOF structures is positive in many application scenarios.^[^ ^21^, ^22^, ^23^, ^24^, ^25^ ^]^ However, MOFs with completely unstable structures can cause great difficulties in their production and preservation.^[^ ^26^ ^]^ Therefore, the construction of MOFs with controllable structural stability is of great significance. For example, Li and co‐workers used ZIFs as metal ion precursors to promote the uniform growth of nanoparticles, and utilized the water resistance instability of ZIFs to construct functional nanocomposites for lithium batteries.^[^ ^27^ ^]^ Zhao et al. constructed a fluorescent sensor for selective monitoring of specific amino acids by utilizing the unstable thiol tolerance of HKUST‐1.^[^ ^28^ ^]^ The Shi group loaded alendronate drugs into UiO‐66 and controlled the precise release of the drug through the poor alkali resistance of UiO‐66.^[^ ^29^ ^]^ Recently, in research on leather manufacturing, it was surprised to find that MOF with controllable structural stability also have enormous potential in the application of chrome‐free tanning.

In the leather industry, tanning is a key process that converts raw skin into leather by coordinating tanning agent molecules with collagen fibers.^[^ ^30^, ^31^, ^32^, ^33^ ^]^ So far, chrome tanning has been the main method in the tanning process, which is attributed to the advantages of high stability, full texture, and easy dyeing of the leather tanned by chrome.^[^ ^34^ ^]^ However, the wastewater containing chromium has caused serious pollution to the environment.^[^ ^35^ ^]^ In addition, after the impact of the COVID‐19, more people have paid attention to the safety and internal quality of leather commodities.^[^ ^36^ ^]^ The Cr^3+^ contained in chromium tanning agents can be oxidized to Cr^6+^, which has carcinogenic effects and is harmful to human health.^[^ ^37^ ^]^ Therefore, the development of green and safe chromium‐free tanning agents is of great significance.

The metal centers in MOFs are self‐masked by organic ligands, which makes them have great potential in the application of leather tanning. Especially, the stable structure of Zr‐MOF makes it more conducive to penetration into collagen fibers. Nevertheless, it has been found that Zr‐MOF could still maintain steady structural after penetrating into collagen fibers in our previous works.^[^ ^38^ ^]^ This phenomenon indicated that a large amount of Zr ions could not bind with collagen fiber, leading to a decrease in the utilization efficiency of Zr ions. Therefore, a Zr‐MOF with adjustable structural stability, which can maintain structural stability during tanning penetration to promoting the penetration performance. When Zr‐MOF permeated uniformly in the collagen fibers, its stability can be altered through simple chemical transformation, allowing MOF fully bind with collagen fibers. This strategy could further achieve controllable permeation and binding of Zr‐MOF.

Herein, DL‐tartaric acid (TA) was utilized as an organic ligand to synthesize a novel zirconium‐based MOF structure (Zr‐TA) through solvothermal method (Inspired by MOF‐801), and the structure was characterized through XRD, SEM, and Virtual modeling (TOPAS). Based on this, continue to convert the ‐OH of TA ligand into ‐COOEt with stronger electron‐withdrawing capacity through esterification reaction, which is used to enhance the electronegativity of the ligand, weaken strength of the metal coordination bond, and regulate the stability of the MOF structure. In other words, this strategy can maintain the structural integrity of MOF during tanning penetration and decompose during crosslinking, thereby maximizing the utilization of Zr ions while improving tanning uniformity. Furthermore, in order to explore the tanning performance of Zr‐TA, the surface morphology, heat resistance stability, and physical properties of the leather tanned by Zr‐TA were also tested. Meanwhile, the functional groups and structural changes of Zr‐TA during the tanning process were monitored by NMR and XRD, and a reasonable tanning mechanism was proposed.

## 2 Results and Discussion

### 2.1 Characterization of Zr‐TA

**Figure 1:**
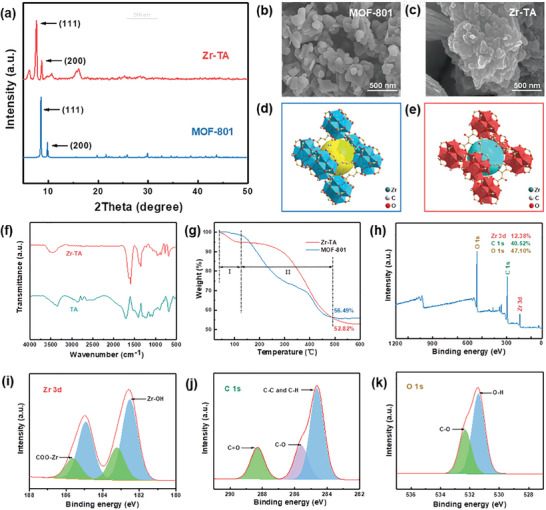
a) XRD patterns, b‐c) SEM images, and d–e) the structural diagrams of MOFs, f) FT‐IR spectra of Zr‐TA and TA, g) TG curves of MOFs, h–k) XPS spectra of Zr‐TA.

Tartaric acid comes from various plants and is also the main organic acid component in wine.^[^ ^39^ ^]^ It is mainly used in food preservation, organic catalysis, biomass refining, and clean energy fields.^[^ ^40^, ^41^, ^42^, ^43^ ^]^ In addition, the organic ligand of MOF‐801 is fumaric acid, and the preparation of tartaric acid is usually obtained through the oxidation reaction of fumaric acid.^[^ ^44^, ^45^ ^]^ It is worth noting that they have similar chemical structures and properties. Therefore, the corresponding MOF structure can also be obtained with TA as a ligand. The test results of powder X‐ray diffraction (PXRD) confirmed this hypothesis. As shown in **Figure** **1**a, the diffraction peaks of Zr‐TA at 7.74° and 8.72° correspond respectively to the (111) and (200) crystal planes of MOF‐801,^[^ ^46^ ^]^ indicating the presence of a crystal structure similar to MOF‐801 in Zr‐TA. Besides, the position of the Zr‐TA diffraction peak signal shifts toward a lower angle. This is because the C2‐C3 bond type of TA is C(sp^3^)‐C(sp^3^), which is longer than the C(sp^2^)‐C(sp^2^) bond length of fumaric acid.^[^ ^47^ ^]^ This means that Zr‐TA has a larger interplanar spacing.

The surface morphology and size of the tanning agent samples were observed by SEM. As shown in Figure 1b,c, compared to MOF‐801, the particle size of Zr‐TA is smaller, with a diameter of ≈100–150 nm. Notably, the Zr‐TA sample showed severe aggregation, which is attributed to the presence of more ‐OH in the TA ligand, leading to the formation of numerous hydrogen bonds between particles and triggering aggregation.^[^ ^48^ ^]^

In addition, the lattice parameters a, b, and c of Zr‐TA are not equal, α, β, γ = 90° and the space group is p n n n (**Table** **1**). These results indicate that the crystal cell of Zr‐TA is an orthogonal system with a configuration of rhombic octahedron, which is different from the regular octahedral structure of MOF‐801 (Figure 1d,e).^[^ ^49^, ^50^ ^]^

**Table 1:** Crystal information of MOFs.

Name	Sp.gr	a[Å]	b[Å]	c[Å]	α, β, γ[°]	V^3^[Å]
Zr‐TA	P n n n	18.482	18.553	16.228	90.0	5564.57
MOF‐801	P n 3	17.931	17.931	17.931	90.0	5765.09

The crystal cell parameter information of MOF‐801 comes from the CCDC crystal database.^[^ ^49^ ^]^

FT‐IR spectra also confirmed the successful synthesis of Zr‐TA. As shown in Figure 1f, the broad peak located between 3180 and 3658 cm^−1^ in Zr‐TA belongs to the stretching vibration peak of the ‐OH, proving the absence of coordination between Zr ions and ‐OH of TA. Meanwhile, the absorption peaks at 1388 and 1583 cm^−1^ belong to carboxyl C‐O symmetric and asymmetric stretching vibrations, respectively, showing a significant red shift compared to the TA band.^[^ ^51^ ^]^ The reason is that Zr ions coordinated with ‐OH of TA, the electron‐withdrawing effect of Zr ions weakened the strength of ligand bonds and reduced the vibration frequency of ‐COOH, indicating the successful connection between Zr ions and the ‐COOH of TA. In addition, the absorption peak at 775 cm^−1^ belongs to the stretching vibration peak of Zr‐O coordination bonds in Zr‐MOFs, confirming the formation of metal–oxygen cluster structures in MOFs.

The thermal stability of Zr‐TA and MOF‐801 was compared by thermogravimetric analyzer. As shown in Figure 1g, the thermal weight loss in the first stage is mainly due to the volatilization of DMF solvent in the pores of the MOF, while the thermal weight loss in the second stage is mainly caused by the collapse of the MOF structure, which is the loss of ligands and the destruction of metal–oxygen cluster.^[^ ^52^ ^]^ After the second stage, the MOF structure completely collapsed and only ZrO_2_ remained. The results indicated that the heat resistance stability of Zr‐TA was higher than MOF‐801, but its residual carbon content is lower. The reason for this phenomenon was that lower zirconium content in Zr‐TA, thereby remaining less residual ZrO_2_ after calcination. This means that under the same tanning conditions, the zirconium utilization rate of Zr‐TA is lower and costs are saved.

The quantitative analysis of the elements contained in Zr‐TA was carried out through XPS testing (Figure 1h). Furthermore, the chemical state and types of chemical bonds of the elements were confirmed through high‐resolution XPS spectra. Among them, Figure 1i shows the high‐resolution XPS spectrum of Zr 3d. The two peaks at 185.0 and 182.6 eV belong to the Zr 3d 3/2 and 3d 5/2 signals of Zr‐TA, respectively. After deconvolution, two sets of double peaks were obtained, which belong to Zr‐OH (182.5 and 184.9 eV) and COO‐Zr (183.2 and 185.6 eV), respectively, further confirming the generation of zirconium oxygen cluster structure (the SBU of MOF) and the successful coordination of Zr ions with the ─COOH of TA ligand.^[^ ^53^ ^]^ Figure 1j,k sequentially displayed the high‐resolution XPS spectra of C 1s and O 1s, capturing the peak signals of C‐C/H (284.6 eV), C‐O (285.6 eV and 532.3 eV), C = O (288.3 eV), and O─H (531.4 eV), confirming the presence of TA ligands and their uncoordinated ─OH, which was consistent with the FT‐IR test results.

### 2.2 Shrinkage Temperature of Tanned Leathers

**Figure 2:**
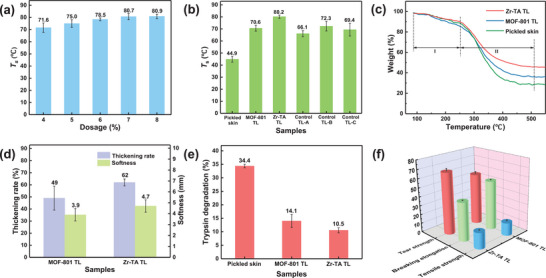
a,b) *T*_s_, c) TG curves of samples, d) the softness and the thickening rate, e) trypsin degradation, and f) the physical properties of MOFs tanned leather.

The dosage of Zr‐TA tanning agent was optimized using *T*_s_ as a reference index. First, the *T*_s_ of 4% to 8% of Zr‐TA tanned leather samples (TL) were recorded. As shown in **Figure** **2**a, with the increase of tanning agent dosage, the *T*_s_ of the leather sample were significantly improved. When the tanning agent dosage was 7%, the leather sample obtained the optimal *T*_s_ of 82 °C (average 80.7 °C). If the tanning agent dosage was further increased, the effect on the *T*_s_ of the leather sample was minimal. Subsequently, under the optimal tanning agent dosage conditions, we compared the *T*_s_ of Zr‐TA and MOF‐801 tanned leather samples (as shown in Figure 2b), and the results showed that the *T*_s_ of Zr‐TA tanned leather samples were remarkably improved compared to traditional Zr‐MOF (MOF‐801). Afterward, the *T*_s_ of tanned leather samples with TA‐Zr complexes (Control TL‐A), Zr‐TA with added difluoric anhydride (Control TL‐B), and Zr‐TA without added propionic anhydride (Control TL‐C) were also compared. The results showed that Zr‐TA tanned leather samples had the best *T*_s_, which not only proves the MOF structure facilitates the permeation of Zr ions into collagen fibers but also indicates that changes in the electronegativity of MOF ligands affect the binding effect of Zr ions with collagen fibers. (Optimization of more tanning conditions, as shown in Figures S1–S5, Supporting Information)

### 2.3 Thermal Stability of Tanned Leathers

The thermogravimetric test results are shown in Figure 2c, the weight loss of the skin sample is mainly divided into two stages. Originally, at temperatures below 250 °C as the first stage, the thermal weight loss is mainly caused by the evaporation of free water in the leather. Then, the range of 250–500 °C as the second stage, and the quality loss in this range is mostly caused by the decomposition of collagen fibers. The results indicate that Zr‐TA tanned leather has higher residual quality and stronger heat resistance stability after two stages of thermal weight loss.^[^ ^54^ ^]^

### 2.4 Physical and Sensory Properties of Tanned Leather

The softness and thickening rate of tanned leather were tested as shown in Figure 2d, the leather tanned with Zr‐TA had a higher thickening rate and softness, improving the problem of traditional zirconium tanned leather with a harder surface (zirconium tanning agent is prone to accumulate on the leather surface, causing over tanning and increased hardness). These indirectly indicate that Zr‐TA has stronger permeability in the collagen of the leather and a more uniform tanning process. Meanwhile, the chemical stability of tanned leather was tested through enzyme degradation experiments (Figure 2e). The results showed that after 24 h of trypsin decomposition, the residual quality of Zr‐TA tanned leather was the highest, indicating that it has the best enzyme degradation resistance. Furthermore, the tear strength, elongation at break, and tensile strength of tanned leather were tested to evaluate its quality and durability. The test results showed that the physical properties of Zr‐TA tanned leather were improved compared to MOF‐801 (Figure 2f), indicating a tighter binding between Zr‐TA and collagen fibers.

### 2.5 Morphology of Tanned Leather

**Figure 3:**
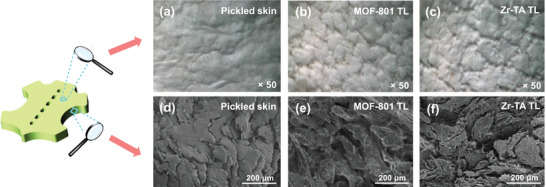
a–c) The ultra‐depth optical microscopy photos of the corresponding grain surface, d–f) the SEM images of samples.

The surface morphology of pickled skin, MOF‐801 tanned skin, and Zr‐TA tanned skin were sequentially photographed by ultra‐depth optical microscopy. As shown in **Figure** **3**a, the surface of the pickled skin is relatively smooth without obvious pores. The reason is that collagen accumulates in the pickled skin without cross‐linking.^[^ ^55^ ^]^ However, MOF‐801 tanned leather showed significant pores and improved surface roughness, which is a surface convergence phenomenon caused by tanning crosslinking (Figure 3b). Fortunately, this phenomenon is more prominent in Zr‐TA‐tanned leather (Figure 3c). It not only generates deep and uniform pores, but also exhibits stronger convergence, proving a more uniform tanning effect and superior cross‐linking strength.

Furthermore, the cross‐section of the leather samples mentioned above was observed through SEM. As shown in Figure 3d, the pickled skin lacks cross‐linking, resulting in a large amount of collagen fiber accumulation on its cross‐section, which is consistent with the observation results of ultra‐depth optical microscopy. On the contrary, MOF‐801 tanned leather exhibits good dispersibility due to the influence of tanning crosslinking, and there are obvious gaps between the collagen fibers bundles (Figure 3e). As expected, Zr‐TA‐tanned leather has stronger dispersibility, exhibiting smaller fiber bundles and denser gaps (Figure 3f). This is because MOF‐801 has a sufficiently stable structure, which limits the distribution of Zr ions in collagen.^[^ ^56^ ^]^ However, the instability of the Zr‐TA is controllable, the Zr ions in its structure are fully utilized to produce a more uniform tanning effect.

### 2.6 Distribution of Tanning Agents

**Figure 4:**
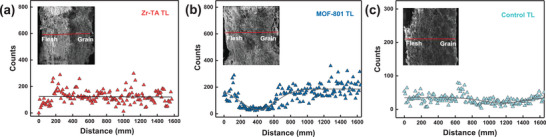
Distribution of Zr elements in MOFs tanned leather. a) Zr‐TA tanned leather, b) MOF‐801 tanned leather, c) unesterified Zr‐TA tanned leather.

The distribution of Zr elements in collagen fibers was investigated through EDS testing. As shown in **Figure** **4**a,b, compared to MOF‐801 tanned leather, The more uniform distribution of Zr elements in Zr‐TA tanned leather proves that Zr‐TA has stronger tanning performance compared to traditional MOFs.^[^ ^57^ ^]^ Surely, to verify the benefits of Zr‐TA esterification on tanning, the distribution of Zr elements in unesterified Zr‐TA tanned leather was compared (Figure 4c). The results showed that the Zr elements content in the unesterified Zr‐TA tanned leather was relatively low, indicating that the impact of esterification on the tanning effect of Zr‐TA cannot be ignored, which is completely consistent with our design.

### 2.7 The Binding Effect Between Zr‐TA and Collagen Fibers

**Figure 5:**
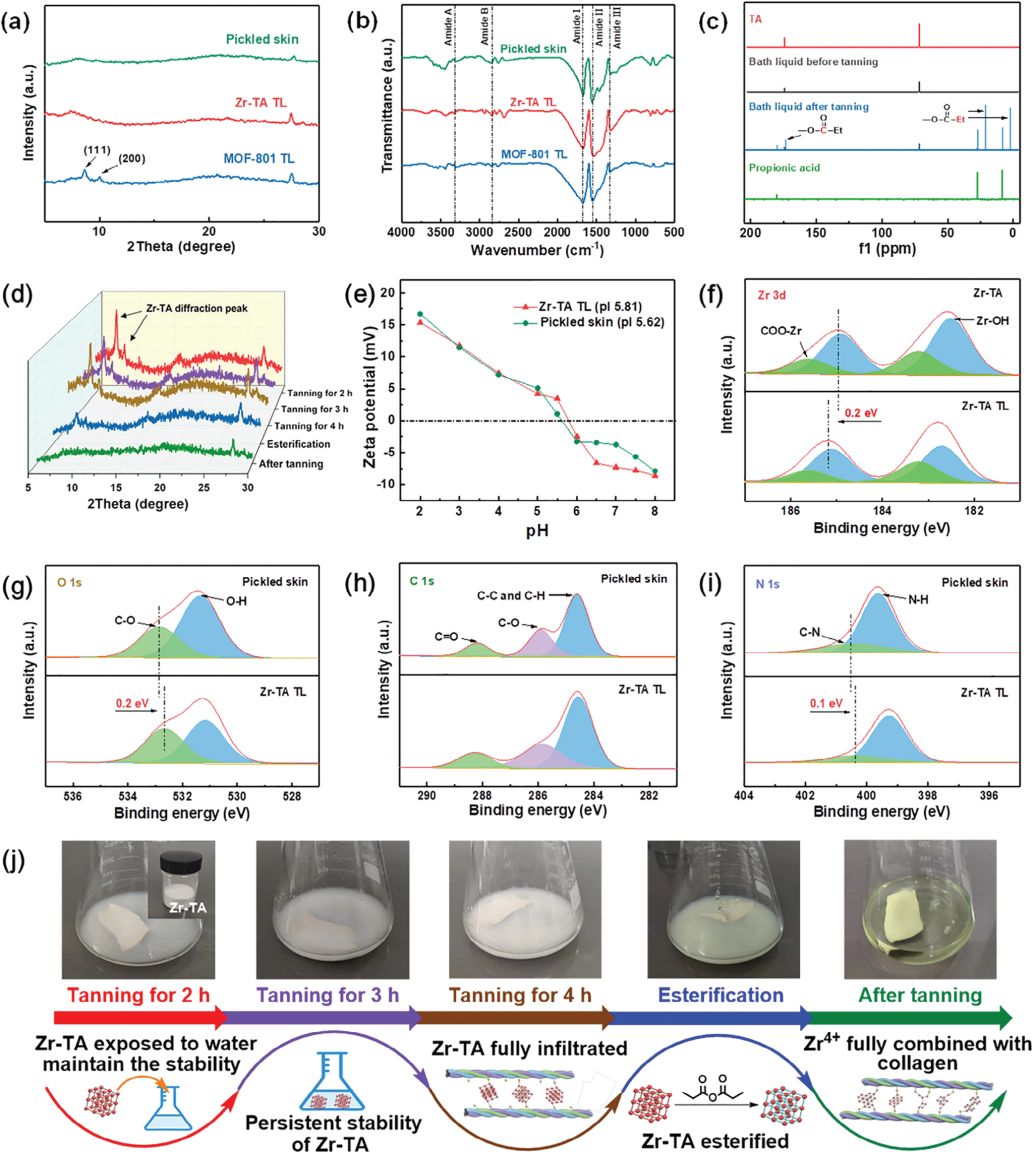
a) XRD patterns, b) FT‐IR spectra of samples, and c) ^13^C NMR of Zr‐TA tanned leather at different tanning stages, and d) XRD patterns of Zr‐TA tanned leather at different tanning stages, and e) Zeta potentials of pickled skin and Zr‐TA tanned leather, and f–i) High‐resolution XPS spectra of Zr‐MOFs, and j) Schematic diagram of Zr‐TA in different tanning stages.

The tanned leather of Zr‐TA and MOF‐801, and pickled skin were characterized by XRD. As shown in **Figure** **5**a, the Zr‐TA tanned leather exhibits diffraction peak signals near 7° and 20°, which is consistent with the pickled skin. They belong to the collagen molecular gaps and amorphous diffuse reflections of collagen fiber structure.^[^ ^58^ ^]^ This means that the tanning effect of Zr‐TA will not cause damage to the triple helix structure of collagen. Meanwhile, this point also can be confirmed by FT‐IR, as shown in Figure 5b, the Zr‐TA tanned leather showed all amide characteristic peaks of collagen fibers, corresponding to the pickled skin spectrum band, proving that the structure of collagen fibers remained intact after tanning.^[^ ^59^ ^]^ Besides, by comparing the XRD pattern of the tanning agent molecules with the tanned leather samples, it was found that MOF‐801 still exhibits significant diffraction peaks in the tanned leather, indicating that MOF‐801 still maintains structural integrity in the collagen fibers, which is consistent with our previous experimental results.^[^ ^38^ ^]^ However, the signal of Zr‐TA disappeared in tanned leather, proving that its structure collapsed in the collagen fibers.

In order to characterize the functional changes of Zr‐TA ligands, the tanning process was reproduced by using D_2_O instead of H_2_O, and characterized the tanning solution before and after tanning by ^13^C NMR (Figure 5c). The results indicated that there were only trace amount of carbonyl carbon signal of TA in the solution during the tanning process. The reason is that a few unprocessed TA was loaded into the Zr‐TA pores and ultimately released during the tanning process. However, a new carbonyl carbon signal peak appears in the ^13^C NMR spectrum of the tanning solution with the addition of propionic anhydride, which is different from the carbonyl carbon signal of TA or hydrolyzed propionic acid.^[^ ^60^ ^]^ Based on the chemical shift and changes in the number of carbon in the fatty region, it can be determined as an ester carbonyl carbon signal, proving the occurrence of esterification reaction.

The tanning process of Zr‐TA was monitored by XRD. As shown in Figure 5d, the diffraction peaks of Zr‐TA stabilized during the tanning infiltration process until the signal disappeared with the addition of propionic anhydride. In brief, the MOF structure of Zr‐TA remains stable during the tanning infiltration process. After MOF Uniformly penetrating into the collagen fibers, Zr ions can fully bind with the collagen fibers by regulating the ligand electronegativity through propionic anhydride promotes the collapse of the MOF structure, which is consistent with the hypothesis (Figure 5j).

Furthermore, the zeta potential of pickled skin and Zr‐TA‐tanned leather was tested. As shown in Figure 5e, the isoelectric point (pl) of Zr‐TA tanned leather is significantly higher than pickled skin. This result demonstrates that Zr ions coordinate with the ‐COOH of collagen fibers, reducing the negative charge of collagen fibers.

**Figure 6:**
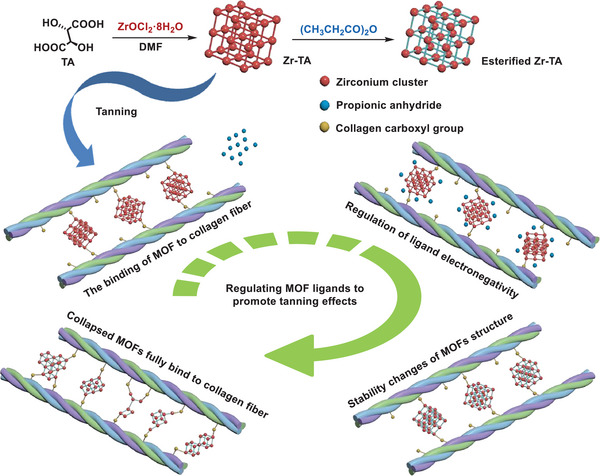
The possible interactions between Zr‐TA and collagen fibers.

The changes in binding energy of samples before and after tanning were detected by XPS. First, the Zr 3d spectra of Zr‐TA and its tanned leather were compared. As shown in Figure 5f, a signal peak of Zr element was captured in the Zr‐TA tanned leather, which corresponds to the Zr‐TA spectrum, proving the successful introduction of Zr element. Furthermore, the C 1s, O 1s, and N 1s spectra between pickled skin and Zr‐TA tanned leather were compared (Figure 5g–i). It is worth noting that the binding energy of the C‐O peak in Zr‐TA tanned leather is reduced by 0.2 eV compared to pickled skin. On the other hand, the O‐H signal in the O 1s spectrum mainly belongs to the collagen fibers ‐COOH, while the O‐H signal peak intensity in Zr‐TA tanned leather is significantly reduced, which is also sufficient to prove the coordination effect between collagen fibers ‐COOH and Zr ions.^[^ ^38^ ^]^ By the way, compared with pickled skin, the binding energy of the C‐N peak in Zr‐TA tanned leather decreased by 0.1 eV, and the intensity of the N‐H peak was weakened, indicating that Zr ions may also have a coordination effect with the ‐NH_2_ on collagen fibers. Based on the above results analysis, we propose a possible tanning mechanism for Zr‐TA (**Figure** **6**).

**Figure 7:**
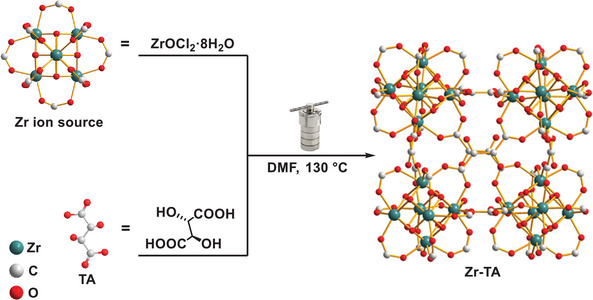
Solvothermal synthesis of Zr‐TA.

## 3 Conclusion

In summary, a strategy based on adjusting the electronic environment of ligands to regulate the structure stability of MOF was disclosed. Among them, a novel Zr‐MOF structure by using TA as ligands was synthesized, which the structure was similar to the MOF‐801. It is worth mentioning that there were two active hydroxyl groups on the Zr‐TA unlike MOF‐801, which could occur the esterification reaction by introduced propionic anhydride. This strategy could controllably change the binding strength between metal center and ligand, achieving the controllable regulation of the structure stability of Zr‐TA. Lastly, it was investigated the modification effect of collagen fibers crosslinked by Zr‐TA with different structure stability in this work. Remarkably, Zr‐TA has a higher zirconium utilization rate and lower cost than traditional Zr‐MOF (MOF‐801), simultaneously Zr‐TA crosslinked collagen fibers possessed better hydrothermal stability, sensory, and mechanical properties. Best of all, this strategy will open up a new pathway for controlling the structural stability and application performance of MOF.

## 4 Experimental Section

### Materials

DL‐tartaric acid (TA), Zirconium oxychloride octahydrate (ZrOCl_2_·8H_2_O), propionic anhydride (C_6_H_10_O_3_), and difluoroacetic anhydride (C_4_H_2_F_4_O_3_) was purchased from Macklin Biochemical Co., Ltd. formic acid(HCOOH), potassium carbonate (K_2_CO_3_), ethanol (C_2_H_5_OH), and N, N‐dimethyl formamide (DMF) were obtained from Energy Chemical Co., Ltd. pickled goatskins (about 0.8 mm thick) were commercially available.

### Synthesis of Zr‐TA

The synthesis conditions of Zr‐TA are shown in Figure S1 (Supporting Information). Briefly, ZrOCl_2_·8H_2_O (5 mmol, 1.61 g) and DL‐tartaric acid (5 mmol, 750 mg) were completely dissolved in DMF. Then, they were thoroughly mixed and transferred to a high‐pressure reaction vessel lined with polytetrafluoroethylene for 12 h at 130 °C. Subsequently, the reaction mixture was cooled to room temperature and a white precipitate was obtained through centrifugation. Afterward, the white precipitate was washed with EtOH and dried at 80 °C for 24 h in vacuum to obtain Zr‐TA (**Figure** **7**).

### Characterization of Zr‐TA

The XRD spectrum of Zr‐TA was obtained through X‐ray diffraction instrument D8 Advance (Bruker, Germany) testing, and its crystal data was obtained through TOPAS (Bruker, Germany). The Fourier transform infrared spectrum was obtained through testing with an infrared spectrometer Nicolet Is10 (Thermo, USA). Element composition, chemical bond types, and chemical valence states of Zr‐TA were analyzed by X‐ray photoelectron spectrometer AXIS SUPRA (Kratos, UK), and its thermal stability was tested by thermogravimetric analyzer TGA‐55 (Discovery, USA). In addition, the microstructure of Zr‐TA was captured by emission scanning electron microscope (Rigaku, S4800).

### Tanning Process

The optimization of the detailed tanning process for Zr‐TA is shown in Figures S1–S4 (Supporting Information). Briefly, traditional zirconium tanning had a lower penetration pH, while Zr‐TA was more suitable for penetration under alkaline conditions. The reason was that in alkaline environments, the hydroxyl groups on the side chains of Zr‐TA were prone to deprotonation and form negative oxygen ions, which have a stronger electron‐donating effect to enhance the ligand electronegativity and promote the stability of MOF structure.

Before tanning, the pickled skin was cut into appropriate sizes and placed in a drum containing NaCl aqueous solution (7 Baume degree, 200% pickled skin weight) for 10 min rotation. Later, the pH of the bath solution in the drum was adjusted to 8 by the alkaline pH regulator K_2_CO_3_. Then, Zr‐TA tanning agent was added to the drum and tanned continuously for 4 h. After that, propionic anhydride was slowly added to adjust the pH to 4 and the drum temperature was raised to 37 °C. Under these conditions, the drum continued to rotate for 1 h in order to change the electronic environment through esterification reaction. Finally, the heating was stopped and the drum was left to stand overnight to promote the full combination of tanning agent and leather. The next day, the leather was removed from the drum and washed with pure water to obtain a leather sample.

### Optimization of the Zr‐TA Dosage

Shrinkage temperature (*T*_s_) was one of the important physical parameters of leather, which was of great significance for the production and use of leather commoditize. Therefore, the optimal dosage of Zr‐TA tanning agent was optimized using *T*_s_ as the indicator. Tanned leather was tested by the leather shrinkage temperature tester (MSW‐YD4, Shaanxi University of Science and Technology, China). Each sample was tested four times simultaneously (samples parallel and perpendicular to the collagen fibers were selected for two repeated tests), and the average value was calculated to reduce testing errors.

### Comparison Experiment of Zr‐TA Tanning

In order to investigate the tanning effect of Zr‐TA, the *T*_s_ of leather samples tanned by traditional Zr‐MOF (MOF‐801) were compared. In addition, the *T*_s_ of leather tanned with zirconium tartrate complex, Zr‐TA and difluoroacetic anhydride, and Zr‐TA without propionic anhydride were compared, which can verify the feasibility of improving the tanning effect through MOF ligand electronegativity regulation strategy.

### Performance of Tanned Leathers

The *T*_s_ of Zr‐TA, MOF‐801, and zirconium tartrate complex tanned leather were tested by the leather shrinkage temperature tester MSW‐YD4. Furthermore, the thermal stability of Zr‐TA and MOF‐801 tanned leather was compared by leather shrinkage temperature tester MSW‐YD4.

The thermal stability of Zr‐TA, MOF‐801 tanned leather, and acid‐washed leather was compared by using a thermogravimetric analyzer TGA‐55.

Field emission scanning electron microscopy was used to observe the surface and cross‐sectional morphology of pickled skin, MOF‐801, and Zr‐TA‐tanned skin samples. On the other hand, the surface particle morphology of pickled leather, MOF‐801, and Zr‐TA tanned leather samples was captured by using a super depth of field microscope (Keyence, Japan).

In order to further understand the dispersion of tanning agents in tanned leather and investigate the uniformity of tanning process, the distribution of Zr‐TA and MOF‐801 in leather collagen was detected by field emission scanning electron microscopy and energy dispersive spectroscopy (EDS). Certainly, in order to verify the effectiveness of the MOF ligand electronegativity regulation strategy, a control tanning experiment of Zr‐TA was conducted in a nonpropionic anhydride bath solution, and energy dispersive spectroscopy (EDS) was used to compare the distribution of Zr‐TA in collagen fibers under different environments of standard tanning and control tanning.

In the process of examining the sensory performance of tanned leather, the softness of tanned leather samples was tested by a leather softness tester NBC‐7124 (TST, China), and the thickness of raw skin and tanned leather samples were measured by a leather thickness tester GB8949 (MZHU China) to calculate the leather thickening rate. At the same time, in order to test the physical and chemical properties of tanned leather, the tensile strength, tear strength, and elongation at break of tanned leather samples were tested by using digital rupture strength tester TX031 (TST, China), and investigated the enzymatic degradation resistance of tanned leather through trypsin degradation experiments.

### The Binding Effect Between Zr‐TA and Collagen Fibers

The interaction mechanism between Zr‐TA and collagen fibers was verified through a series of experiments. Among them, the protein structure of the tanned skin sample was analyzed by XRD and FT‐IR, which can confirm whether the tanning agent has caused damage to the collagen fiber structure of the skin. Meanwhile, the elemental content and chemical bond types of raw skin and tanned leather were analyzed by X‐ray photoelectron spectrometer to confirm the binding mode between tanning agents and collagen fibers. The isoelectric point of leather samples was tested by laser particle size analyzer (Mastersizer, UK). In addition, the successful conversion of ligand groups was verified by analyzing the tanning solution before and after tanning by NMR 600 MHz(Bruker, German).

## Conflict of Interest

The authors declare no conflict of interest

## Supporting information



Supporting Information

## Data Availability

The data that support the findings of this study are available in the supplementary material of this article.
