# Supplementary material for: Controllable Strategy of Metal–Organic Framework Structural Stability: Regulation of Ligand Electronegativity by Esterification
Source: Adv Sci (Weinh). 2024 Dec 5;12(4):2413853. doi: 10.1002/advs.202413853 (PMC11789593; doi:10.1002/advs.202413853)
Supplement: Supplementary file 1 — Supporting Information [file ADVS-12-2413853-s001.docx]

MOFs with Controllable Structural Stability by Esterification

Guanjie Huang ^a, b, c^, Jianzhong Ma ^a, b, c*^, Jie Chen ^a, b, c^, Wenbo Zhang ^a, b, c^, Qianqian Fan ^a, b, c^, Buxing Han^d^

^a^College of Bioresources Chemical and Materials Engineering, Shaanxi University of Science & Technology, Xi’an 710021, Shaanxi, China

^b^Xi’an Key Laboratory of Green Chemicals and Functional Materials, Shaanxi University of Science & Technology, Xi’an 710021, Shaanxi, China

^c^National Demonstration Center for Experimental Light Chemistry Engineering Education, Shaanxi University of Science & Technology, Xi’an 710021, Shaanxi, China

^d^Beijing National Laboratory for Molecular Sciences, CAS Key Laboratory of Colloid and

Interface and Thermodynamics, Institute of Chemistry, Chinese Academy of Sciences, Beijing

100190, China.

Email: majz@sust.edu.cn

**Supporting Information**

Table of Contents:

Contents

1. **Optimization of Zr-TA synthesis conditions 2**
2. **Optimization of tanning conditions 3**
3. **Simulated calculation of TOPAS 5**
4. **^13^C NMR of tanning liquid 6**
5. **Reference8**
6. **Optimization of Zr-TA synthesis conditions**

**Table S1 Optimization of Zr-TA synthesis conditions**

| Entry | Zirconium source | Solvent | Temperature (℃) | Reaction time (h) | Yield (%) |
| --- | --- | --- | --- | --- | --- |
| 1 | ZrCl_4_ | DMF | 120 | 6 | 17.1 |
| 2 | Zr(SO_4_)_2_·4H_2_O | DMF | 120 | 6 | 33.4 |
| 3 | Zr(NO_3_)_4_·5H_2_O | DMF | 120 | 6 | 58.8 |
| 4 | ZrOCl_2_·8H_2_O | DMF | 120 | 6 | 73.5 |
| 5 | ZrOCl_2_·8H_2_O | C_2_H_5_OH | 120 | 6 | N.D. |
| 6 | ZrOCl_2_·8H_2_O | CH_3_OH | 120 | 6 | N.D. |
| 7 | ZrOCl_2_·8H_2_O | THF | 120 | 6 | 58.6 |
| 8 | ZrOCl_2_·8H_2_O | DMSO | 120 | 6 | 66.5 |
| 9 | ZrOCl_2_·8H_2_O | H_2_O | 120 | 6 | N.D. |
| 10 | ZrOCl_2_·8H_2_O | DMF | r.t. | 6 | N.D. |
| 11 | ZrOCl_2_·8H_2_O | DMF | 60 | 6 | 28.4 |
| 12 | ZrOCl_2_·8H_2_O | DMF | 80 | 6 | 55.1 |
| 13 | ZrOCl_2_·8H_2_O | DMF | 100 | 6 | 69.3 |
| 14 | ZrOCl_2_·8H_2_O | DMF | 110 | 6 | 74.2 |
| 15 | ZrOCl_2_·8H_2_O | DMF | 130 | 6 | 77.6 |
| 16 | ZrOCl_2_·8H_2_O | DMF | 140 | 6 | 77.9 |
| 17 | ZrOCl_2_·8H_2_O | DMF | 130 | 4 | 75.8 |
| 18 | ZrOCl_2_·8H_2_O | DMF | 130 | 8 | 78.1 |
| 19 | ZrOCl_2_·8H_2_O | DMF | 130 | 10 | 79.4 |
| **20** | **ZrOCl_2_·8H_2_O** | **DMF** | **130** | **12** | **81.7** |
| 21 | ZrOCl_2_·8H_2_O | DMF | 130 | 13 | 80.4 |
| 22 | ZrOCl_2_·8H_2_O | DMF | 130 | 24 | 80.9 |

As shown in Table S1, this study screened the sources of zirconium, solvents, temperature, and reaction time in the Zr-TA synthesis process. The optimal synthesis conditions are shown in entry 20.

1. **Optimization of tanning conditions**

In addition to the optimization of tanning agent Zr-TA tanning agent dosage mentioned in the main text, this work also screened the tanning pH, tanning binding temperature, tanning time and types of alkaline additives in the tanning process^1-4^ (6% dosage).

**Figure S1**

As shown in Figure S1, Zr-TA has poor tanning effect and permeability under acidic conditions, resulting in uneven tanning. This is because the MOF structure of Zr-TA is prone to decomposition. In contrast, Zr-TA tanning under alkaline conditions can effectively enhance *T*_s_, but the pH should not be too high, otherwise it will lead to the hydrolysis of skin collagen.

**Figure S2**

As shown in Figure S2, appropriate heating is beneficial for increasing the *T*_s_ of Zr-TA tanned leather. The reason is that heating is conducive to the occurrence of esterification reaction. When the temperature is increased to 37 ℃, further increasing the temperature has little effect.

**Figure S3**

As shown in Figure S3, the optimal tanning time is around 4 h. It is worth noting that the tanning efficiency of Zr-TA is extremely high. With just 1 h of Zr-TA tanning, the *T*_s_ of the leather can approach 70 ℃.

**Figure S4**

As shown in Figure S4, this work attempted to use 5 kinds of alkalis as additives in the Zr-TA tanning process to regulate the tanning pH. Among them, NaOH is too alkaline, causing damage to the collagen structure of the skin, resulting in lower *T*_s_, while NaHCO_3_ is too alkaline, which is not conducive to the esterification reaction. On this basis, we attempted Na_2_CO_3_ and K_2_CO_3_ with slightly stronger alkalinity and found that K_2_CO_3_ achieved the best effect (*T*_s_ reached 78.4 ℃). In addition, organic alkali KOCH_3_ was continued to be attempted, which is less effective than K_2_CO_3_.


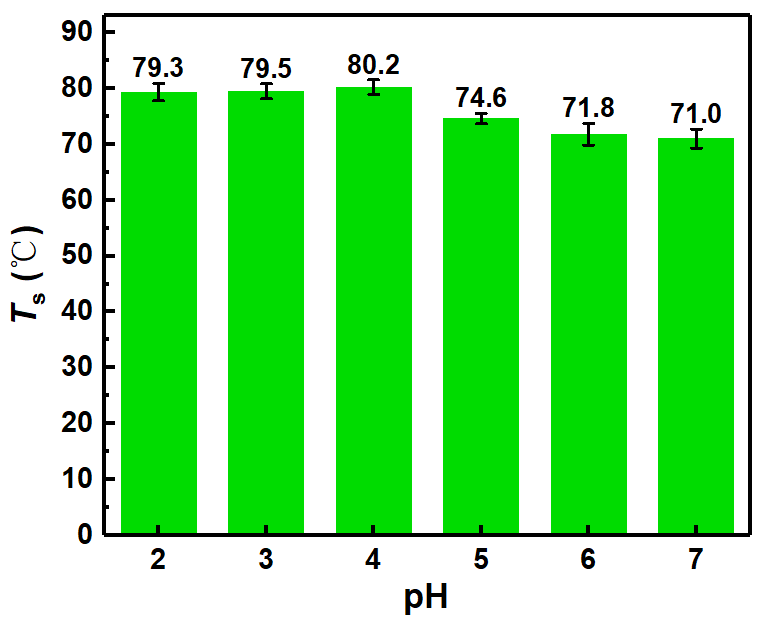


**Figure S5**

Tanning experiments were conducted using different doses of propionic anhydride (6% Zr-TA), and the pH value of the tanning mixture was controlled using five control groups with pH values of 7, 6, 5, 3, 2 by adding different doses of propionic anhydride. The results showed that as the degree of Zr-TA esterification increased, the *T*_s_ of tanned leather gradually increased. However, excessive esterification has a relatively small impact on the *T*_s_ of tanned leather.

1. **Simulated calculation of TOPAS**

**Figure S6**

Subsequently, the PXRD spectra of Zr-TA were refined and analyzed using TOPAS (Figure S5), and detailed crystal parameters of Zr-TA were obtained^5,6^ (Table S2).

**Table S2. Main parameters of processing refinement.**

| **compound** | **Zr-TA** |
| --- | --- |
| **sp. gr.** | P n n n |
| **a (Å)** | 18.482 |
| **b (Å)** | 18.553 |
| **c (Å)** | 16.228 |
| **α, β, γ (°)** | 90.0 |
| **V (Å^3^)** | 5564.569 |
| **2θ-interval** | 5°-53° |
| **no. of reflection** | 587 |
| **no. of refined parameters** | 113 |
| **R_wp_ (%)** | 4.52 |
| **R_p_ (%)** | 3.72 |
| **R_exp_ (%)** | 3.65 |
| ***ꭓ*^2^** | 1.23 |
| **R_Br_ (%)** | 1.29 |

1. **^13^C NMR of tanning liquid**

**
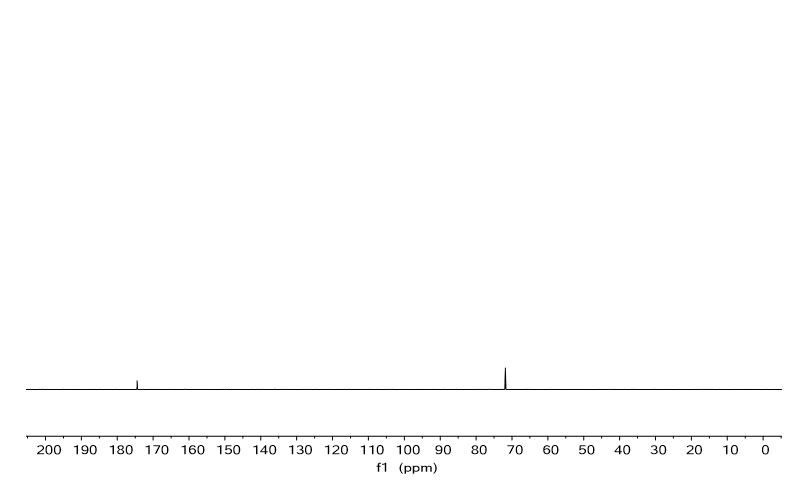
**


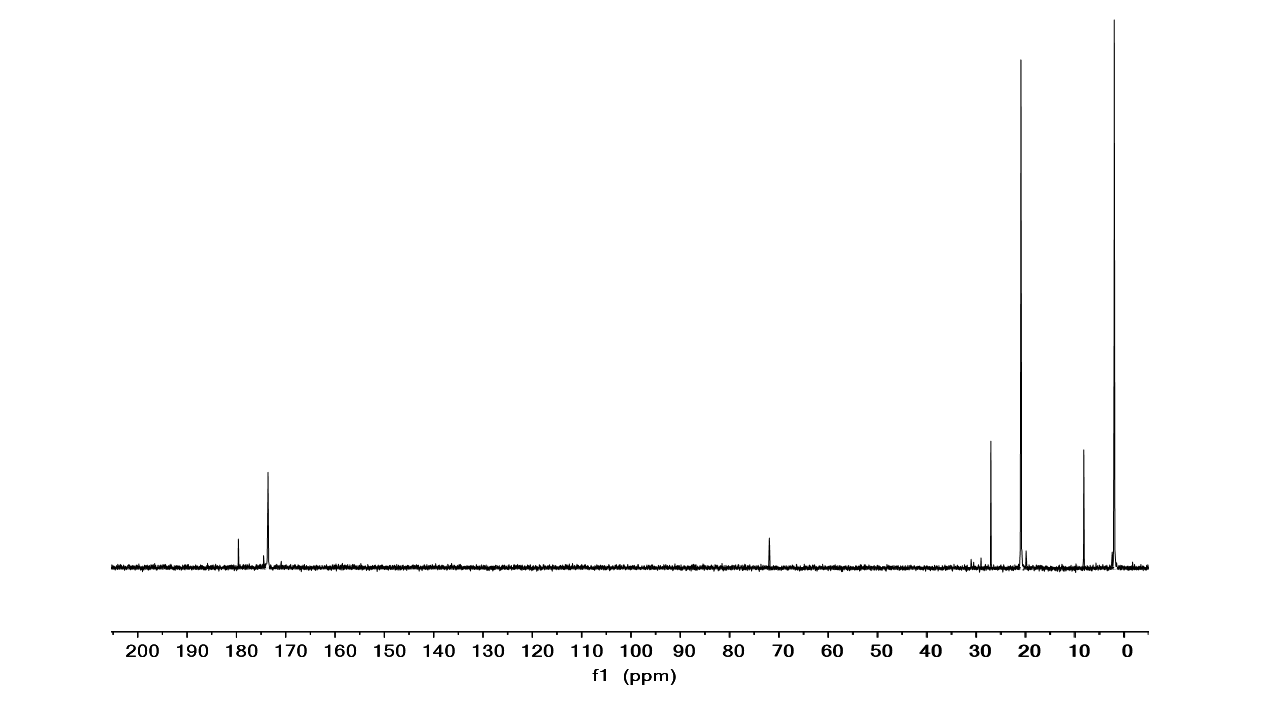


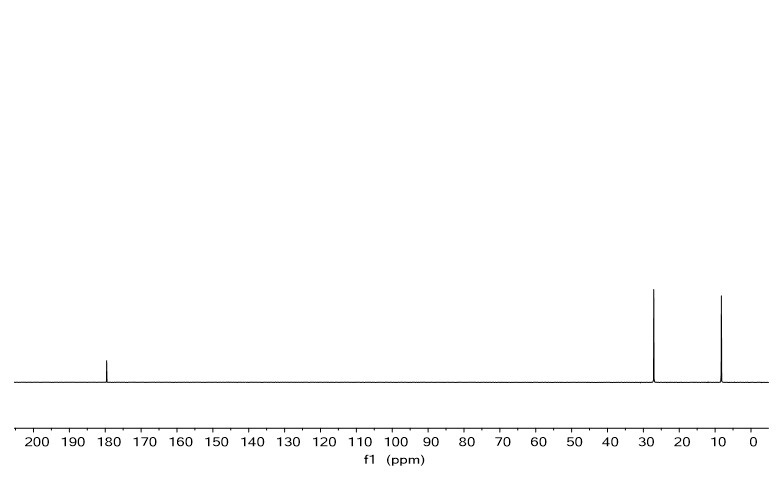


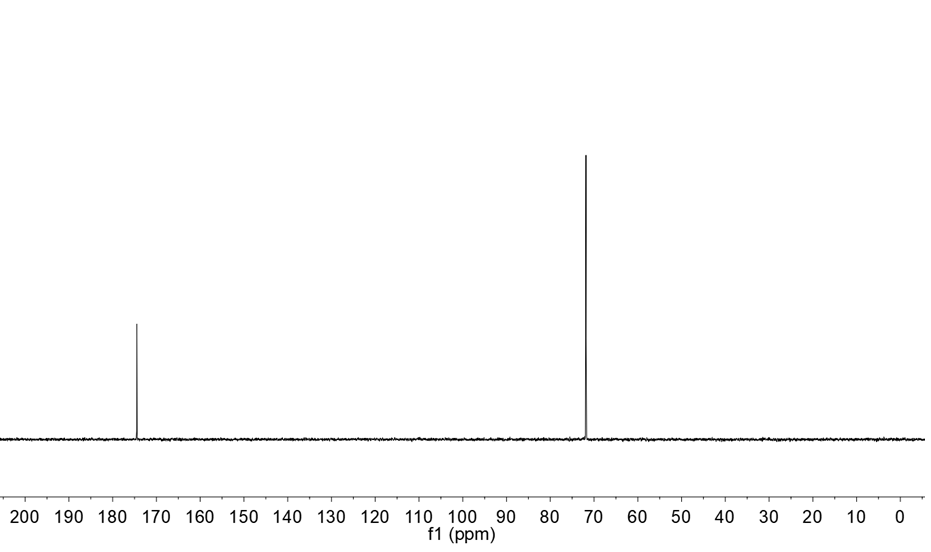


1. **Reference**
2. L. Zhang, Q. S. Cheng, C. H. Wang, C. P. Huang, W. Lin, *J. Clean. Prod*. **2024**, *457*, 142464.
3. H. Pan, G. L. Li, R. Q. Liu, X. D. Wang, *Appl. Surf. Sci*. **2017**, *426*, 376.
4. X. C. Wang, L. Han, S. G. Qu, X. G. Dang, L. X. Feng, *Int. J. Biol. Macromol*. **2024**, *268*, 131682.
5. D. G. Gao, P. P. Wang, J. B. Shi, L. Fan, J. Z. Ma, *J. Clean. Prod*. **2019**, *229*, 1102.
6. H. Jahangiri, M. L. Öveçoğlu, *Matt. Lett*. **2016**, *178*, 193.
7. J. Zhang, J. L. Zhang, X. G. Lu, L. Q. Li, C. Zhang, *J. Eur. Ceram. Soc*. **2018**, *38*, 5471.
